# The Role of Green Tea on the Regulation of Gut Microbes and Prevention of High-Fat Diet-Induced Metabolic Syndrome in Mice

**DOI:** 10.3390/foods12152953

**Published:** 2023-08-04

**Authors:** Huiling Mei, Jin Li, Shujing Liu, Anburaj Jeyaraj, Jing Zhuang, Yuhua Wang, Xuan Chen, Qijun Yuan, Xinghui Li

**Affiliations:** 1 College of Resources and Environmental Sciences, Nanjing Agricultural University, Nanjing 210095, China; 2019203030@njau.edu.cn; 2 College of Tourism and Economic Management, Nanchang Normal University, Nanchang 330032, China; lijin@ncnu.edu.cn; 3 International Institute of Tea Industry Innovation for the Belt and Road, Nanjing Agricultural University, Nanjing 210095, China; liushujing395@gmail.com (S.L.); geneanbu1986@gmail.com (A.J.); zhuangjing@njau.edu.cn (J.Z.); wangyuhua@njau.edu.cn (Y.W.); chenxuan@njau.edu.cn (X.C.); 4 Northern Tea Germplasm Resource Center, Rizhao 276808, China; ajjyqj@126.com

**Keywords:** green tea, metabolic disease, *Akkermansia*, gut bacteria, tea polyphenols

## Abstract

Green tea is a popular non-alcoholic beverage consumed worldwide and has been shown to be beneficial for human health. However, further exploration is needed to fully understand its function in reducing obesity and regulating gut microbes. Here, we investigated the modulatory effects of green tea and its functional components on high-fat diet (HF)-induced metabolic alterations and gut microbiota in obese mice. Our results showed that 1%, 2%, and 4% of green tea promotes weight loss, with the 2% and 4% groups exhibiting distinct gut microflora clusters compared to the HF group. These results were comparable to those observed in the tea polyphenols (TPP)-treated group, suggesting the TPP in green tea plays a crucial role in body weight control and gut microbiota regulation. Additionally, 32 bacteria were identified as potential obesity markers via 16S rRNA gene sequencing. The 16SrDNA gene is a chromosomal gene present in all bacterial species, highly conserved in structure and function, that can reflect the differences between different taxa. The 16S rRNA-based analysis revealed that *Akkermansia*, a gut-beneficial bacteria, significantly increased in the TPP group.

## 1. Introduction

Obesity is a significant health issue in modern society with more than 500 million obese people and 1.4 billion overweight individuals in the world [1,2]. It is associated with various metabolic diseases, such as hyperglycemia, hyperlipidemia, and high blood pressure. Abdominal obesity and insulin resistance, accompanied by elevated blood glucose metabolism, elevated serum triglycerides, elevated blood pressure, high-density lipoprotein cholesterol, and increased low-density cholesterol, are the obesity indicators [3,4]. Mounting evidence has shown a link between intestinal microflora and obesity [5]. Obese microbiota has been found to increase dietary energy harvest and favor weight gain and fat deposition [6,7]. Disturbance of gut microbiota in high fat (HF)-induced obesity and diabetes mice leads to metabolic endotoxemia, inflammation, and associated disorders by increasing intestinal permeability [8,9]. Additionally, gut microbiota may play an active role in insulin resistance development [10]. Therefore, the intestinal microflora is closely related to the formation and development of metabolic syndrome and its related symptoms.

Green tea is a popular non-alcoholic beverage known for its functional effects on humans and animals with functional components, such as tea polyphenols (TPP), caffeine (Caf), and L-theanine (Thea) [11,12]. Two major mechanisms have been identified in the functional effects of green tea: (1) decreasing the absorption of lipids and proteins in the intestine, thereby reducing calorie uptake [13,14], and (2) activating AMP-activated protein kinase via TPP that is bioavailable in the liver, skeletal muscle, and adipose tissues [15]. Animal models and human studies have shown that green tea reduces body weight, alleviates metabolic syndrome, and prevents diabetes and cardiovascular diseases [16,17]. TPP mainly accumulates (90–95%) in the large intestinal lumen where gut microbial communities enzymatically break down the accumulated polyphenolic structures into a series of low-molecular-weight phenolic metabolites [18]. The green tea components interact and affect the gut microbe population. For example, fermented green tea extract can reduce Firmicutes to Bacteroidetes and Bacteroides to Prevotella in the large intestine of obese mice [19,20]. Polyphenols have been shown to increase the content of *Akkermansia*, a gut-beneficial bacterium that is negatively correlated with various excessive fat-related diseases, such as inflammatory bowel disease, obesity, type 2 diabetes, and appendicitis [21,22]. This study investigated the role of green tea and its functional components (TPP, Caf, and Thea) on the regulation of intestinal microflora and reducing obesity.

## 2. Materials and Methods

### 2.1. Preparation of Green Tea Infusion

Suchazao tea cultivar leaves were collected from Zhongshanling tea garden (Nan-jing, China) and made into green tea after deactivation of enzymes and torrefaction treatment [23,24]. With reference to the standardized concentrations for tea beverage preparation, 1, 2, and 4 g green tea were mixed with 100 mL distilled water at 100 °C, which was maintained for 30 min by using a water bath. Total TPP content was quantified by spectrophotometric method based on Folin phenol and the ninhydrin assay as described by GB/T 8313-2018, and Caf and Thea contents were detected in the brewed tea via high-performance liquid chromatography (HPLC) (Alliance 2695, Waters, America, Milford). To evaluate the effectiveness of the main components of green tea, concentration of TPP in the second experiment was set to 6 g per liter, where the concentration was equivalent to the total concentration of TPP in 4% green tea. Constitute profile of the 4% green tea is shown in Table 1.

### 2.2. Animal and Treatments

Healthy male C67BL/6J mice (10~12 g, 3~4 weeks old) (Permission number: 201604129) were obtained from Animal Centre, Yangzhou University, and kept in a growth chamber in the Animal Centre, Nanjing Agricultural University, in a controlled environment with a temperature range of 20~26 °C, relative humidity of 40~70%, light intensity of 250 Lux, and a photoperiod of 14 h light followed by 10 h darkness. They were given one week to acclimatize to their surroundings before starting the experiment. To investigate the effect of green tea on obesity, 35 mice were randomized into 5 groups and labeled as LF, HF, HF+1%, HF+2%, and HF+4%. The mice in the LF and HF groups were fed a low-fat (10% fat calories) and high-fat (60% fat calories) diet, respectively, while mice in HF+1%, HF+2%, and HF+4%groups were fed an HF diet with corresponding 1, 2, and 4% green tea infusions, respectively, for a period of 8 weeks. Also, to assess the effect of major components of green tea on the regulation of the gut microbiota, 35 mice were randomized into 5 equal groups and labeled HF + TPP, HF + Thea, HF + Caf, LF, and HF groups. According to the proportion of the 3 major components in green tea, mice in the HF + TPP, HF + Thea, and HF + Caf groups were fed an HF diet with 6.0 gL^−1^ TPP, 1.5 gL^−1^ Thea, and Caf, respectively. Feeding and water supply (with or without treatments) were performed ad libitum granting the mice equal opportunity and access to feed and water. The amount of water intake and the change in body weight were recorded daily. All protocols for the animal experiments complied with the Nanjing Agricultural University guideline for care and use of laboratory animals.

### 2.3. Tissue Preparation and Biomarker Assessments

Oral glucose tolerance test (OGTT) was performed to determine the glucose tolerance level of the mice. After 12 h starve treatment, the blood serum of mice was collected from the tail to detect the fast blood glucose (FBG) level. The glucose was then given to the mice by oral gavage (2 g/kg, 10% solution) for OGTT. Thereafter, the mice were fasted for 12 h, anesthetized, and sacrificed by cervical dislocation after peripheral blood collection from the ophthalmic vein. Histological studies were conducted on the liver and small intestine. Tissues were fixed with 0.04 g/mL^−1^ paraformaldehyde in 0.01 M phosphate buffer for 24 h and then processed for paraffin inclusion. Serial paraffin sections (4 mm) were stained with haematoxilin-eosin (HE) followed by light microscopy examination (KingMed Diagnostics, Nanjing, China). The number of goblet cells per small intestinal villus was counted [24]. Alanine aminotransferase (ALT) (C009), aspartate aminotransferase (AST) (C010), triglyceride (TG) (A110), total cholesterol (TC) (A111), LDL (low-density lipoprotein) (A113), HDL (high-density lipoprotein) (A112), and free fatty acids (FFA) (A045) (Njjcbio, Nanjing, China) in serum were determined by using commercially available kits according to the manufacturer’s instructions. Serum contents of mono-cytechemo attractant protein-1 (MCP-1) (60224M), insulin (INS) (60207M) (Rigor, Beijing, China), tumornecrosisfactor-α (TNF-α) (E21030M), and LPS (Lipopolysaccharide) (E21618M) (Aogene, Nanjing, China) were detected using ELISA kits.

### 2.4. Gut Microbiota Classification by Next Generation Sequencing (NGS)

Total DNA was extracted from 0.2 g of fresh fecal samples collected from each mouse following the manufacturer’s instructions for DNA extraction kit (MoBio, Carlsbad, CA, USA). The DNA amplification was performed in 25 µL reaction volumes using primer sequences (520F/802R) targeting the V4 region of the 16S rRNA gene [25]. The purified amplicons were pooled in equimolar concentrations and employed for library construction using the NEBNext^®^UltraTM DNA Library Prep Kit for Illumina (New England Biolabs, Hitchin, UK). The final quality and concentration of the libraries were assessed using Agilent 2100 Bioanalyzer Instruments (Agilent Technologies, Santa Clara, CA, USA) and determined with KAPA Library Quantification Kits (Kapa Biosystems, Wilmington, MA, USA). All library preparations for sequencing were performed on the Illumina MiSeq platform. Sequencing reads were assigned to each sample according to their unique barcodes. Sequences were analyzed with QIIME 1 (Quantitative Insights into Microbial Ecology) software package and UPARSE pipeline [26]. The reads were filtered with QIIME quality filter [27]. The sequences retained for each sample, referred to as clean paired sequences, were analyzed following the UPARSE pipeline to pick up operational taxonomic units (OTUs) through the creation of OTU table. Sequences with quality scores lower than 0.5, lengths shorter than 200 nt, and singletons were discarded, and then retained sequences were assigned to OTUs at 97% similarity. Chimeras were filtered. Representative sequence was selected for each OTU, and using Ribosomal Database Project (RDP) classifier [28], the taxonomic data were assigned to each representative sequence. To reveal distinct clustering of microbiota composition for each treatment group, UniFrac-based principal coordinate analysis (PCoA) was performed using Rlanguage (Version 3.3.1). Resultant Venn diagram was then drawn using Bioinformatics and Evolutionary Genomics (http://bioinformatics.psb.ugent.be/webtools/Venn/, accessed on 1 May 2023).

### 2.5. Statistical Analysis

Data are shown as mean ± SEM. Differences were analyzed for statistical significance using one-way analysis of variance (ANOVA) with Turkey’s multiple comparison test to compare samples (Graph Pad Prism, San Francisco, CA, USA). Differences in body weight were assessed using two-way ANOVA. Values of *p* < 0.5 were considered significant. Next-generation sequencing analysis was assessed using PERMANOVA to check significant differences between microbial assemblages, and packages of DESeq2 were used for the identification of differentially abundant microbial features.

## 3. Results

### 3.1. Effect of Green Tea on Body Weight- and Fat-Associated Biochemical Indices of HF-Induced Obese Mice

**Figure 1:**
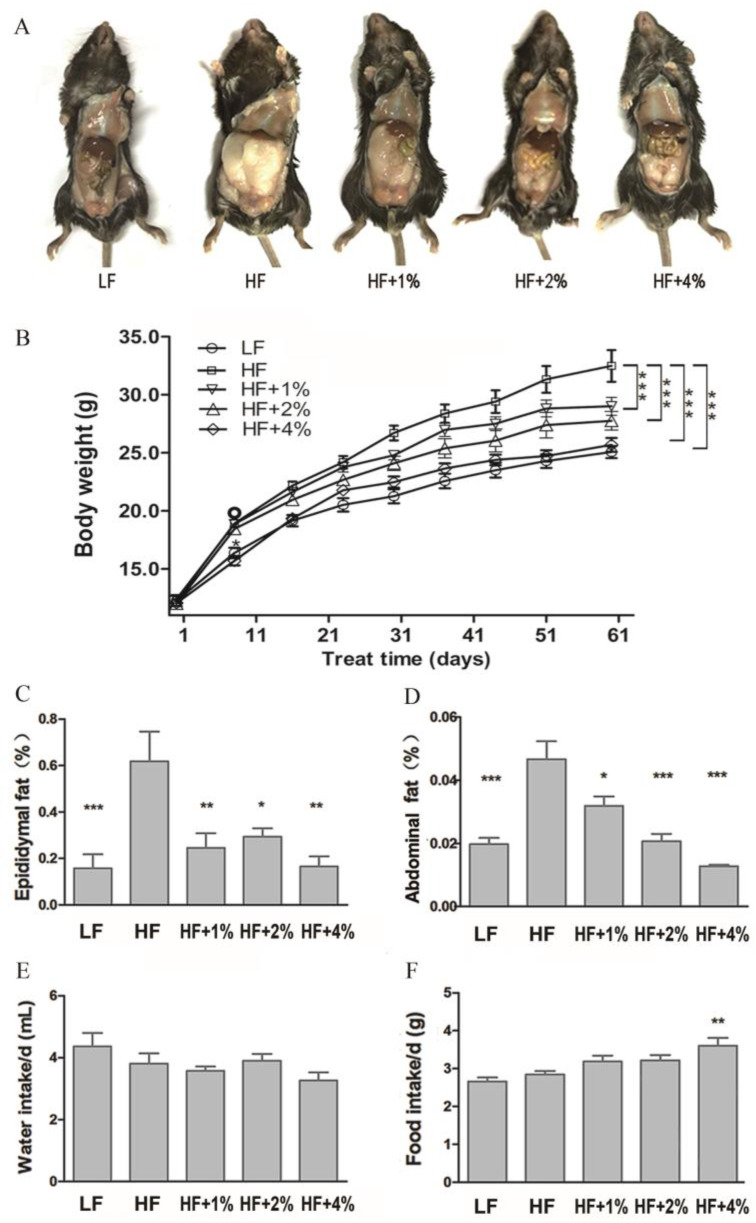
Green tea prevents HF-induced obesity in mice. Image of hypodermic fat in different groups of mice (**A**). The body weight (**B**), epididymal fat (**C**), and abdominal epididymal fat (**D**) in the different groups of mice. Water (**E**) and food (**F**) intake in different groups of mice. LF represents low-fat-fed mice and HF represents high-fat-fed mice. * *p* < 0.05; ** *p* < 0.01; *** *p* < 0.001.

**Figure 2:**
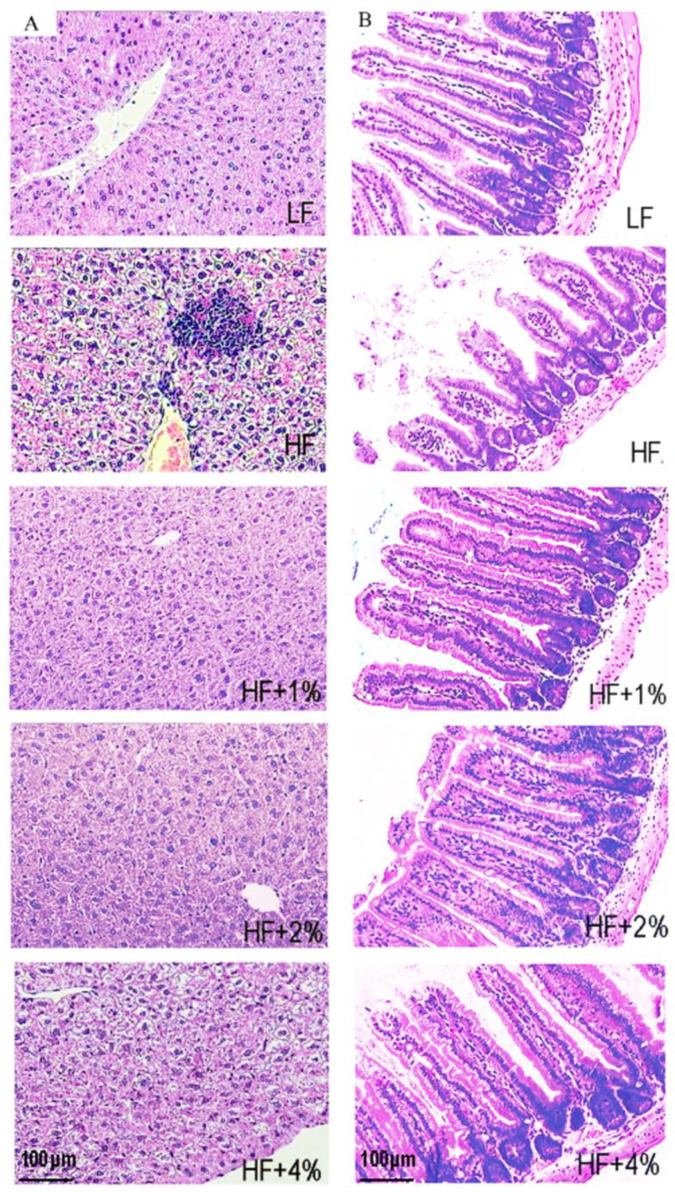
Green tea improves obesity-caused hepatic dysfunction and intestinal barrier integrity. The liver cell (**A**) and intestinal villus (**B**) in different groups of mice with HE staining (100×).

After 8 weeks of feeding, compared to the LF group, the weight, subcutaneous, epididymal, and abdominal fat of the HF group mice significantly increased, indicating the successful construction of a high-fat diet-induced obesity model. However, there was no significant difference in diet and water intake between the HF group and the LF group (Figure 1). To assess the impact of green tea on obesity, we fed the mice with LF, HF, HF with 1% green tea (HF+1%), HF with 2% green tea (HF+2%), and HF with 4% green tea (HF+4%) diets. We found that the body weight, hypodermic, epididymal, and abdominal fat were significantly higher in the HF group compared to the LF group mice, whereas the HF diet combined with green tea reduced the mice’s body weight and fat accumulation (Figure 1A–D). The water intake of the mice in all the groups had no significant difference (Figure 1E). The HF+4%group had the highest food intake compared to the LF and other HF groups (Figure 1F), suggesting the water or food intake did not affect the body weight or fat accumulation. Generally, the biochemical indices were higher in the LF group, and fat accumulation was higher in the HF group, but these biochemical indices were decreased in the tea-consumed mice (Table 2). A histological investigation of the liver and small intestine showed that swelling cells with mild lymphocytes were in the portal area of the liver cells in the HF group mice, while dense and regularly arranged cells without the hydroncus phenomenon were in the green tea-treated mouse liver cells (Figure 2A). Additionally, ALT and AST were decreased in the groups treated with HF together with green tea (Table 2), suggesting that green tea has the potential to repair liver damage caused by obesity. In the small intestine, HF-induced shortening and loss of villus and swelling on the intestinal glands were alleviated in the green tea treatment groups. (Figure 2B.) The inflammatory biomarkers TNF-α and MCP-1 were significantly altered in the HF group and the 2% and 4% green tea-treated groups (Table 2).

### 3.2. Effect of Green Tea on HF-Induced Gut Dysbiosis

**Figure 3:**
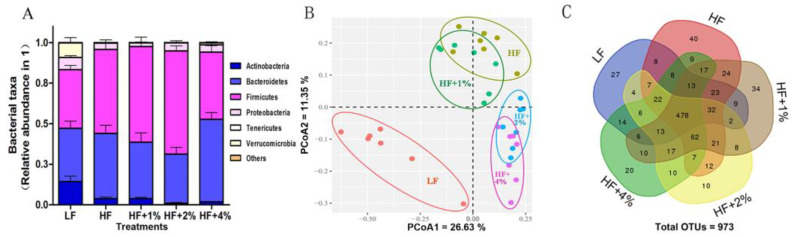
Green tea prevents fat-induced gut dysbiosis. The gut microbiota composition by Illumina MiSeq platform-based analysis of V4 region (**A**). PCoA separation between LF and HF, HF and HF+2%, as well as HF and HF+4%tea treatment groups. Rvegan package was used with 999 per mutation (**B**). Venn diagrams showing the specific bacterial amount (**C**).

To determine the effect of green tea on mice gut microbiota, we examined the composition of intestinal microbiota via NGS. After the removal of low-quality reads, a total of 2,028,825 bacterial 16S rRNA gene sequences were obtained in all the samples. The number of retained bacterial 16S rRNA gene sequences per sample varied between 21,062 and 77,969. A total of 32 bacterial phyla were identified in all samples of these, with Firmicutes (10.30–81.42%) and Bacteroidetes (8.65–65.96%) being dominant and Thermotogae (0–0.01%) and Chloroflexi (0–0.01%) being the least. Additionally, five more minuscule phyla were noticed in most samples (Figure 3A). The clusters of intestinal microflorae were identified in different groups of mice (Figure 3B). PCoA including PCoA1 and PCoA2 were calculated as 26.63 and 11.35%, respectively (Figure 3B). The HF+1% green tea group showed mild separation from the HF group, while the HF+2% and HF+4%green tea groups showed significant separation and were significantly separated from the HF group (Figure 3B). A Venn diagram presented the specific bacterial microorganisms that were altered by HF and green tea intake (Figure 3C). The total number of OTUs of the LF, HF, HF+1%, HF+2%, and HF+4% groups were 672, 782, 769, 711, and 712, respectively. When comparing with the HF group, the HF groups with 1%, 2%, and 4% green tea altered 212 (99 newly appeared and 113 disappeared), 213 (70 newly appeared and 143 disappeared), and 263 (96 newly appeared and 167 disappeared) OTUs, respectively. The OTUs HF+1%, HF+2%, and HF+4% shared with HF mice were 670, 641, and 616, respectively (Figure 3C). The results indicated that the intake of HF with 4% green tea drastically altered the population of intestinal microflora compared to the HF group.

### 3.3. Influence of TPP, Thea, and Caf on Intestinal Bacteria and Obesity-Related Indices of HF-Induced Obese Mice

**Figure 4:**
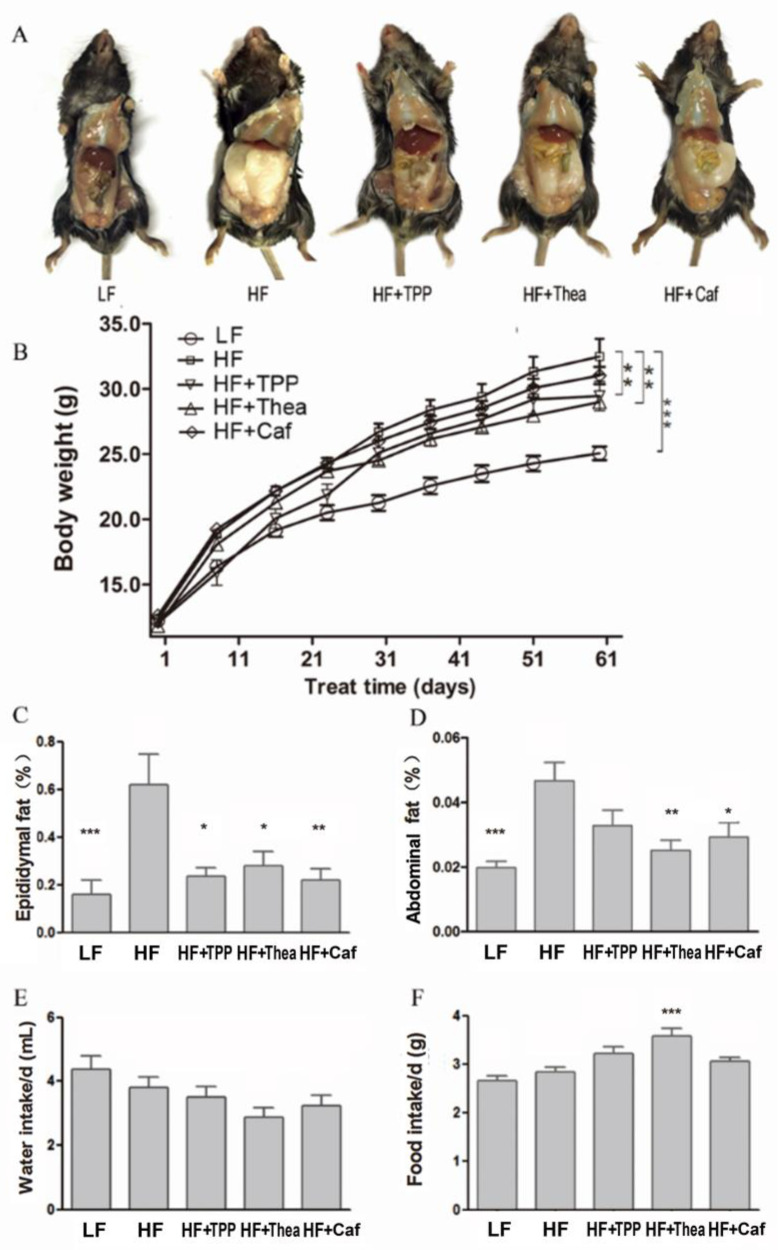
Components of green tea reduce obesity. Image of abdominal fat in mice (**A**). The body weight changes of mice during the 8-week intervention of TPP, caffeine, and L-theanine (**B**). Epididymal fat in green tea-treated mice (**C**) and in the HF mice (**D**). Water (**E**) and food (**F**) intake in different groups of mice. All the other treatments are compared with HF group (*n* = 7). * *p* < 0.05; ** *p* < 0.01; *** *p* < 0.001.

**Figure 5:**
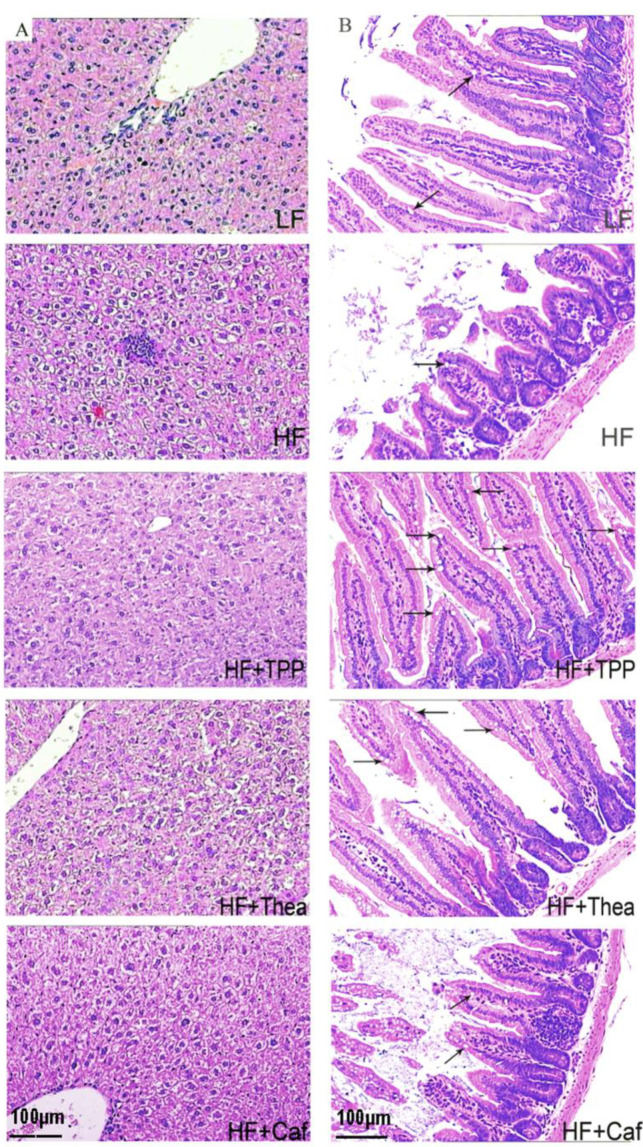
Components of green tea improve obesity-caused hepatic dysfunction and intestinal barrier integrity. The liver cell (**A**) and intestinal villus (**B**) in mice via HE staining (100×). Arrows indicate goblet cells.

After 8 weeks of treatment, the TPP and Thea-treated group showed significant reductions in body weight and fat accumulation compared to the HF group (Figure 4A–D). There was no significant impact on water intake among the mice in all the groups (Figure 4E). The HF + Thea group had the highest food intake and reduced body weight (Figure 4F). TPP treatment significantly reduced the FFA, fat ratio, TG, HDL, and LDL levels (Table 3). The results suggest that TPP plays a critical role in reducing obesity. Morphological analysis of the liver under various tea component treatments were shown in Figure 5A. Swelling and inflammation were observed in the liver cells of HF group mice, and these were relatively less in the Thea, Caf, and TPP groups. The observation in the TPP group can be attributed to the fact that the cells were closely arranged, partially accounting for the reduction in swelling and inflammation (Figure 5A). The HF group had a relatively short and excised villi surface area compared to the LF group. However, the TPP, Caf, and Thea treatments increased the intestinal villi length and surface area (Figure 5B). The villus height, crypt depth, and goblet cell count were significantly higher in the TPP, Thea, and Caf groups compared to the HF and LF groups (Table 4).

### 3.4. Influence of TPP, Thea, and Caf on Gut Dysbiosis of HF-Induced Mice

**Figure 6:**
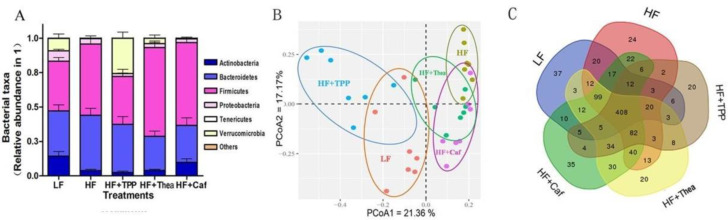
Components of green tea reduce obesity by modulating disrupted microbiota. Relative abundance of bacterial taxa (**A**). PCoA showing the distinct clustering of different components of tea groups (**B**). Venn diagrams showing the specific bacterial amount (**C**). All the other treatments are compared with HF group (*n* = 7).

**Figure 7:**
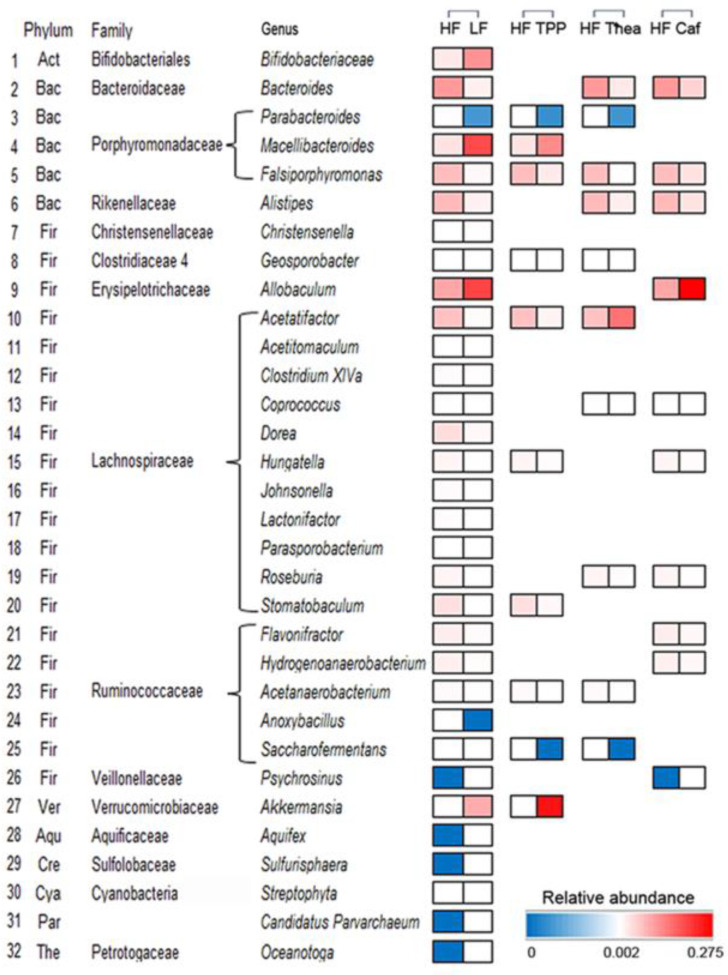
Biomarkers of gut microbes with significant differences between LF and HF groups. The colored part indicates a significant difference from the HF group, while the color depth indicates the relative content of gut microbes. Act-Actinobacteria; Bac-Bacteroidetes; Fir-Firmicutes; Ver-Verrucomicrobia. Aqu-Aquificae; Cre-Crenarchaeota; Cya-Cyanobacteria; Chl-Chloroplast; Par-Parvarchaeota; The-Thermotogae.

The TPP significantly increased Verrucomicrobia and decreased the Firmicutes population in the gut microbiota, respectively. But no significant impact was noticed in HF, Thea, and Caf groups (Figure 6A). The PCoA analysis revealed five distinct groups in the gut microbiota (Figure 6B). The principal coordinates PCoA1 and PCoA were 21.36% and 17.17%, respectively. The clustering of the Thea and Caf groups was partly separated from the HF group, but the TPP cluster was completely separated from HF and closer to the LF cluster (Figure 6B). The OTU of the functional components was investigated to determine their impact on reducing obesity. The total OTUs of the HF + TPP, HF + Thea, and HF + Caf groups were 621, 792, and 821, respectively. The TPP, Thea, and Caf treatments altered 332 (85 newly appeared and 247 disappeared), 221 (115 newly appeared and 106 disappeared), and 232 (135 newly appeared and 97 disappeared) OTUs, respectively. The OTUs of the HF + TPP, HF + Thea, and HF + Caf groups were 536, 677, and 686, respectively (Figure 6C), and the largest number of OTUs was altered by TPP. Among the LF and HF groups, the Student’s t-test revealed 32 significantly different genera of microbiota that were associated with obesity. The effects of TPP, Thea, and Caf on these microbiotas were evaluated, and each functional component significantly altered 10 genera (Figure 7). Overall, the results indicate that green tea intake has a positive correlation with anti-obesity by altering some genera of microbiota.

## 4. Discussion

In this work, we investigated the effect of 1%, 2%, and 4% green tea on body weight and gut microflora dysbiosis and found that green tea with such concentrations contributed to weight loss in HF mice. However, 4% of green tea had the most remarkable effect on controlling body weight, regulating gut microflora, and performing without hepatotoxicity (Table 2). It is worth noting that the high concentration of EGCG (1500 mg/kg) used in the previous study resulted in hepatotoxicity in mice [29,30], which is much higher than our concentration. Therefore, intake of 4% green tea is recommended for the regulation of intestinal microflora.

A large proportion of TPP is converted into simple compounds and metabolites by colon and intestinal microbial enzymes [31,32]. The Bacteroidetes and Firmicutes are the main groups of biomarkers involved in the metabolisms of undigested food remnants, including dietary fiber and polyphenols [33]. Firmicutes were significantly higher in the HF mice compared to the LF mice; in contrast, the Firmicutes/Bacteroidetes ratio decreased in the TPP-treated mice, which is supported by many studies showing that TPP significantly decreases the Firmicutes/Bacteroidetes ratio [34,35,36]. Suppression of Firmicutes by TPP and their metabolites favors Bacteroidetes in the gut of mice, indicating that TPP has a modulatory effect on the composition of the intestinal microbiota. This result is closely associated with previous reports conducted by Higdon and Frei, [32]. But Janssens et al. [37] reported that long-term intake of tea EGCG and Caf does not affect the human gut microbiota. This controversial report may be due to differences in the concentration of the functional components used in these two studies.

A total of 32 obesity-associated biomarkers were identified, including Firmicutes (20 genera) and Bacteroidetes (5 genera). Liu et al. [38] identified 30 key microorganisms that respond to tea, including *Alistipes*, *Akkermansia*, *Bacteroides,* and *Allobaculum*. Our study found that the addition of TPP increased *Akkermansia* abundance in HF mice, which is consistent with previous research indicating that TPP, cranberry, chlorogenic acid, and grape also increase the colonization of *Akkermansia* in the gut [39,40,41,42]. We observed a significant increase in small intestinal goblet cells in TPP-treated mice, which secrete mucins, a food source for *Akkermansia* development [43]. Polyphenols increase intestinal mucus secretion which is the food source of *Akkermansia* and TPP does not alter mucingene (*Muc2*) expression in the jejunum, suggesting that *Akkermansia* is not simply due to increased host’s mucin production [44,45]. Therefore, the potential mechanism of TPP to increase *Akkermansia* needs further investigation.

L-theanine treatment also altered several genera of biomarkers, including disappearing *Saccharofermentans*, like the effect of TPP. Thea with tannins in goat fodder reduces the amount of *Saccharofermentans* in the goats’ rumen [46]. The Thea treatment also altered populations of *Acetatifactor*, *Bacteroides*, and *Alistipes*, with *Acetatifactor* being higher in the Thea group and lower in the TPP group. Additionally, Caf altered the population of the *Allobaculum* genera, which was significantly decreased in the HF mice, and its population density was positively correlated with plasma HDL concentrations of hamsters [47,48]. Mice with barriers to a natural immune response showed a dysbiosis microbiota, reflected by the increase in some *Bacteroidetes* species, including *Alistipes* and *Bacteroides* [49]. *Alistipes* and *Bacteroides* were also overrepresented in carcinoma patients [50,51]. In this study, both *Alistipes* and *Bacteroides* had a positive correlation with body weight, which is significantly increased in the HF group and decreased in the Thea or Caf treatments.

We assessed the effects of three different functional components on the regulation of the intestinal bacterial population and found that TPP played a significant role in specific bacterial population regulation compared to Thea and Caf. At present, the main mechanisms of tea polyphenols in reducing fat and weight loss are as follows: inhibiting the activity of enzymes related to fat synthesis, promoting fatty acid oxidation, and increasing energy consumption in the body while inhibiting appetite and reducing nutrient absorption [52]. Green tea catechins mixed with caffeine have been proposed as adjuvants for maintaining or enhancing energy expenditure and for increasing fat oxidation in the context of the prevention and treatment of obesity [53]. Caf regulated the obesity-associated biomarkers, including *Allobaculum*, *Psychrosinus,* and *Flavonifractor*, while both Thea and Caf regulated *Bacteroides*, *Alistipes*, *Coprococcus,* and *Roseburia*. These components play a complementary role with TPP in maintaining the intestinal flora population and improving the effect of high-fat-induced obesity.

A combination of green tea extract with isomalto-oligosaccharides and tea powder extract can enhance the relative abundance of some probiotics, such as *Bifidobacteriaceae* and *Lactobacillus*, respectively [39]. However, our findings indicate that green tea and its functional components did not support probiotic proliferation. Chen et al. [54] also reported that Fuzhuan brick tea could not enhance *Lactobacillus*.

In summary, we observed a distinct clustering pattern of gut microbiota in mice fed an HF and green tea infusion diet. A 4% green tea infusion significantly increased intestinal microflora in obese mice and reduced high-fat diet-induced metabolic alterations by preventing metabolic disorders associated with the disrupted microflora. Additionally, we identified 32 genera, including *Akkermansia*, *Saccharofermentans*, *Acetatifactor*, *Bacteroides*, *Alistipes*, *Allobaculum,* and *Falsiporphyromonas*, as HF-associated biomarkers. Among these, TPP significantly increased *Akkermansia* and played the most important role in restoring the bacterial community essential for anti-obesity stimulation. Our study suggests that green tea intake is an effective alternative for HF-induced disorders with manipulation of gut microbial communities.

## Figures and Tables

**Table 1:** Constitute profile of green tea and TPP.

Component (gL^−1^)	EC	EGC	ECG	EGCG	C	GC	GCG	CG	TPP	Caf	Thea
4% green tea	0.23	0.61	0.69	1.91	0.07	0.34	0.31	0.18	6.07	1.52	1.50
6 gL^−1^ TPP	0.15	0.01	0.55	2.35	0.01	0.22	0.36	0.14	6.00	-	-

Note: EC, (-)-epicatechin; EGC, (-)-epigallocatechin; ECG, (-)-epicatechin gallate; EGCG, (-)-epigallocatechin gallate; C, (-)-catechin; GC, (-)-gallocatechin; GCG, (-)-gallocatechin gallate; CG, (-)-catechin gallate; TPP, total tea polyphenols; Caf, caffeine; Thea, L-theamine; -, not detected.

**Table 2:** Biochemical levels in mice serum and liver by green tea.

Treatment	LF	HF	HF+1%	HF+2%	HF+4%
TC (mmol/L)	3.76 ± 0.14 ***	6.05 ± 0.34	5.77 ± 0.30	6.18 ± 0.14	4.85 ± 0.17 **
HDL (mmol/L)	2.00 ± 0.27	1.73 ± 0.18	2.50 ± 0.21 *	2.08 ± 0.10	2.25 ± 0.01
LDL (mmol/L)	0.84 ± 0.12 ***	2.16 ± 0.25	1.66 ± 0.08 *	1.93 ± 0.08	1.49 ± 0.06 **
FBG (mmol/L)	4.87 ± 0.19 ***	7.67 ± 0.20	6.44 ± 0.38 *	4.17 ± 0.30 ***	7.01 ± 0.46
INS (mIU/L)	11.62 ± 1.35	9.69 ± 1.18	16.07 ± 2.39 *	17.18 ± 1.40 *	13.18 ± 0.83
ALT (Kar U)	4.83 ± 1.67 *	11.92 ± 2.43	4.89 ± 1.38 *	2.83 ± 1.01 **	1.95 ± 1.00 **
AST (Kar U)	148.00 ± 12.21	148.20 ± 4.54	90.22 ± 8.09 **	120.30 ± 18.39	111.20 ± 12.99 *
LPS (μg/L)	207.90 ± 12.38	245.90 ± 12.84	239.10 ± 21.94	234.00 ± 11.44	237.90 ± 8.12
TNF-α (ng/L)	243.60 ± 23.51 *	309.10 ± 17.32	299.50 ± 8.29	309.00 ± 19.94	275.80 ± 6.95 *
MCP-1 (ng/L)	254.60 ± 33.97 *	193.90 ± 15.58	222.50 ± 37.57	294.50 ± 42.64 **	186.10 ± 48.32
TG (mmol/g pro)	0.13 ± 0.01 **	0.23 ± 0.02	0.20 ± 0.02	0.13 ± 0.01 **	0.18 ± 0.02
FFA (mmol/g pro)	0.10 ± 0.01	0.11 ± 0.01	0.07 ± 0.01*	0.06 ± 0.02 **	0.06 ± 0.01 *

Note: All the other treatments are compared with HF group. Data obtained from three replicate experiments are shown as mean ± SEMs. * *p* < 0.05; ** *p* < 0.01; *** *p* < 0.001. LF, low-fat diet; HF, high-fat diet; HF+1%, high-fat diet with 1% green tea infusion; HF+2%, high-fat diet with 2% green tea infusion; HF+4%represent, high-fat diet with 4% green tea infusion.

**Table 3:** Biochemical levels of mice in serum and liver by functional components.

Treatment	LF	HF	HF + TPP	HF + Thea	HF + Caf
TC (mmol/L)	3.76 ± 0.14 ***	6.05 ± 0.34	5.09 ± 0.39	4.54 ± 0.32 **	5.59 ± 0.30
HDL (mmol/L)	2.00 ± 0.27	1.73 ± 0.18	2.75 ± 0.16 **	2.35 ± 0.19	2.36 ± 0.18
LDL (mmol/L)	0.84 ± 0.12 ***	2.16 ± 0.25	1.56 ± 0.10 *	1.53 ± 0.07 *	1.61 ± 0.27 *
FBG (mmol/L)	4.87 ± 0.19 ***	7.67 ± 0.20	7.93 ± 0.24	8.02 ± 0.36	8.10 ± 0.21
INS (mIU/L)	11.62 ± 1.35	9.69 ± 1.18	10.17 ± 0.90	10.07 ± 2.07	12.79 ± 1.29
ALT (Kar U)	4.83 ± 1.67 *	11.92 ± 2.43	12.55 ± 1.39	13.80 ± 0.82	11.30 ± 1.15
AST (Kar U)	148.00 ± 12.21	148.20 ± 4.54	70.55 ± 9.18 ***	84.82 ± 9.73 ***	103.1 ± 10.39 *
LPS (μg/L)	207.90 ± 12.38	245.90 ± 12.84	186.00 ± 8.61 *	241.34 ± 15.66	224.36 ± 16.95
TNF-α (ng/L)	243.60 ± 23.51 *	309.10 ± 17.32	250.97 ± 11.29 *	284.53 ± 21.73	292.21 ± 16.61
MCP-1 (ng/L)	254.60 ± 33.97 *	193.90 ± 15.58	197.21 ± 19.30	270.79 ± 42.42	247.65 ± 36.67
TG (mmol/g pro)	0.13 ± 0.01 **	0.23 ± 0.02	0.14 ± 0.01 **	0.17 ± 0.02	0.19 ± 0.03
FFA (mmol/g pro)	0.10 ± 0.01	0.11 ± 0.01	0.06 ± 0.01 **	0.09 ± 0.01	0.06 ± 0.01 **

Note: All the other treatments are compared with HF group. Data obtained from three replicate experiments are shown as mean ± SEMs. * *p* < 0.05; ** *p* < 0.01; *** *p* < 0.001. LF, low-fat diet; HF, high-fat diet; HF, high-fat diet; HF+TPP, high-fat diet with 6g/L TPP infusions; HF+Thea, high-fat diet with 1.5 g/L Thea infusions; HF+Caf, high-fat diet with 1.5 g/L Caf infusions.

**Table 4:** Biochemical levels in functional components of treated mice.

Treatment	LF	HF	HF + TPP	HF + Thea	HF + Caf
Goblet cell/villus	5.36 ± 1.51	2.80 ± 0.33	27.67 ± 4.59 ***	24.29 ± 7.08 ***	10.44 ± 3.00
Villus height (μm)	347.50 ± 29.07	289.60 ± 29.71	390.50 ± 33.02 *	411.30 ± 22.99 *	248.70 ± 14.56
Crypt depth (μm)	134.40 ± 4.65 ***	90.55 ± 5.64	133.40 ± 11.19 ***	118.30 ± 4.52 *	119.80 ± 4.78 **

Note: All the other treatments are compared with HF group. Data obtained from three replicate experiments are shown as mean ± SEMs. * *p* < 0.05; ** *p* < 0.01; *** *p* < 0.001. LF, low-fat diet; HF, high-fat diet; HF+TPP, high-fat diet with 6g/L TPP infusions; HF + Thea, high-fat diet with 1.5 g/L Thea infusions; HF+Caf, high-fat diet with 1.5 g/L Caf infusions.

## Data Availability

Data are contained within the article.
